# Ruling Factors in Cinnamaldehyde Hydrogenation: Activity and Selectivity of Pt-Mo Catalysts

**DOI:** 10.3390/nano11020362

**Published:** 2021-02-01

**Authors:** Marta Stucchi, Maela Manzoli, Filippo Bossola, Alberto Villa, Laura Prati

**Affiliations:** 1 Chemistry Department, University of Milano, Via C. Golgi 19, 20133 Milano, Italy; alberto.villa@unimi.it (A.V.); laura.prati@unimi.it (L.P.); 2 Department of Drug Science and Technology and NIS Centre for Nanostructured Interfaces and Surfaces, University of Turin, Via P. Giuria 9, 10125 Torino, Italy; maela.manzoli@unito.it; 3 Istituto di scienze e tecnologie chimiche “Giulio Natta” (SCITEC) CNR, Via C. Golgi 19, 20133 Milano, Italy; filippo.bossola@scitec.cnr.it

**Keywords:** molybdenum, cinnamaldehyde, cinnamaldehyde hydrogenation, carbon supported Mo, bimetallic Pt structure, selectivity

## Abstract

To obtain selective hydrogenation catalysts with low noble metal content, two carbon-supported Mo-Pt bimetallic catalysts have been synthesized from two different molybdenum precursors, i.e., Na_2_MoO_4_ and (NH_4_)_6_Mo_7_O_24_. The results obtained by X-ray photoelectron spectroscopy (XPS) and transmission electron microscopy (TEM) combined with the presence and strength of acid sites clarified the different catalytic behavior toward cinnamaldehyde hydrogenation. After impregnating the carbon support with Mo precursors, each sample was used either as is or treated at 400 °C in N_2_ flow, as support for Pt nanoparticles (NPs). The heating treatment before Pt deposition had a positive effect on the catalytic performance. Indeed, TEM analyses showed very homogeneously dispersed Pt NPs only when they were deposited on the heat-treated Mo/C supports, and XPS analyses revealed an increase in both the exposure and reduction of Pt, which was probably tuned by different MoO_3_/MoO_2_ ratios. Moreover, the different acid properties of the catalysts resulted in different selectivity.

## 1. Introduction

The development of green and sustainable processes is directly connected to the discovering of new heterogeneous catalytic systems capable of selectively transforming bio-derived organic molecules [1]. Decisive factors in the development of sustainable chemical processes are efficiency and selectivity. In this context, the catalytic reduction of carbonyl compounds using H_2_ is a green method to obtain high-added value alcohols for the production of fine chemicals [2]. Many highly active homogeneous or heterogeneous catalysts based on noble metals have been developed for this purpose [3]. Noble metals such as Ru, Pt, and Pd are among the best candidates for catalytic hydrogenation processes [4]. However, they are not the best option considering availability and increasing price. On the other hand, they can be coupled with less precious and active metals, which can improve their performance together with allow using them in a minor extent. Among non-noble transition metals, molybdenum has already been reported as a possible auxiliary low cost catalyst for hydrogenation. For example, molybdenum carbides have been studied in depth as low-cost catalysts for the hydrogenation of levulinic acid to γ-valerolactone [5]. Moreover, Hoang-Van and Zegaoui reported the effects of MoO_3_ on the catalytic properties of Pt for the selective hydrogenations of acrolein and allyl alcohol [6], whereas Wang et al. [7] reported the effect of Mo on the acidity of Pt/TiO_2_ catalyst. Mo-based catalysts synthesized by different approaches have been shown to present different activities. This is not a novelty in heterogeneous catalysis, but the reason remains still unclear. Indeed, the different behavior has been normally explained in terms of different Strong Metal Support Interaction (SMSI) or residual chemicals [8,9].

**Figure 1:**
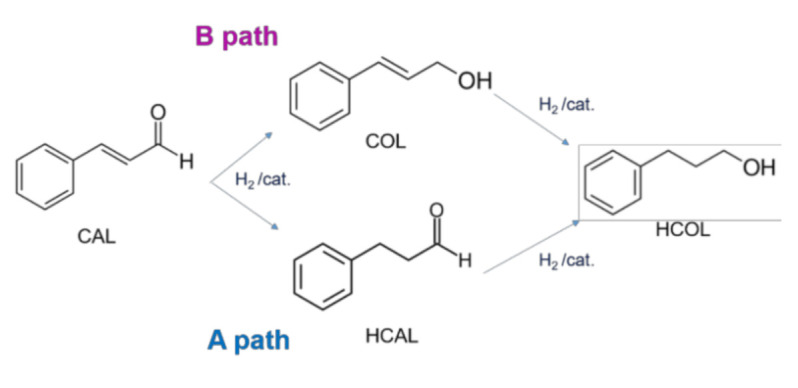
Cinnamaldehyde hydrogenation pathway.

On the basis of these findings, here, we investigated the effect of two different Mo salt precursors in the preparation of Pt-Mo catalysts, which will be tested in the selective hydrogenation of cinnamaldehyde. The hydrogenation of α,β-unsatured aldehydes to the corresponding unsaturated alcohols is often studied as a model reaction, considering that the development of selective catalysts for this class of reactions is challenging, since the simultaneous presence of the carbon–carbon double bond and the carbonyl group [10]. Cinnamaldehyde (CAL) is the most investigated model compound for discriminating the catalytic selectivity for C=C or C=O hydrogenation. Possible pathways are reported in Figure 1.

Moreover, the reaction presents an industrial interest as both hydrocinnamaldehyde (HCAL) or cinnamyl alcohol (COL) are very important intermediates for the synthesis of many fine chemicals, perfumes, and pharmaceuticals [11]. HCAL was found to be an essential intermediate in the preparation of a drug used in the treatment of HIV. COL is one of the most used products in perfumery chemicals [12]. On the other hand, also hydrocinnamyl alcohol (HCOL) is desired, as it represents an important chemical in the pharmaceutical market and cosmetic industry [13]. The strategic importance of this reaction lies in the development of very selective catalysts that are able to direct the hydrogenation toward the desired product. Ma et al. [14] completely hydrogenated CAL to HCOL by Pt supported on carbon nanotubes (CNTs) with 80% of selectivity, but only after 12 h of reaction, and in many other cases, HCOL is produced but with low selectivity [15]. Considering the selectivity of the process, some researchers pointed out a possible role of the metal–support interaction [2,16], with the charge transfers between the support and the metallic phase that increased the selectivity toward COL higher with electron-rich active sites [17,18]. However, it was also found that electron-deficient Au NPs show high selectivity to COL in such reactions [19]. Again, other research works report that Lewis acids or metallic promoters are beneficial for enhancing selectivity to COL, because the electropositive metal species on the surface act as electrophilic or Lewis sites for the adsorption and activation of the C=O bond [10].

To be active and selective for cinnamaldehyde hydrogenation, Pt has been also modified with other metals: Mahata et al. [20] reported the effect of Fe and Zn promotion. In that case, the addition of these metals to Pt was found to improve both the activity and the selectivity to COL, due to the creation of new sites for the activation of the aldehydic group. Wang et al. [21] studied the effect of Cu on Pt, proving that Pt-Cu/SiO_2_ was more selective toward COL than monometallic Pt/SiO_2_, due to the increase in the amount of the Pt^0^ on the surface, which is derived from the interaction between Pt and Cu. To the best of our knowledge, there are only two recent papers reporting Pt modified by Mo in cinnamaldehyde hydrogenation [22,23]. In the first by Wang et al. [22], SBA-15 was modified with Mo_2_N nanoparticles and then used for Pt deposition. A synergistic effect between Pt and Mo_2_N was observed, and both activity and selectivity to COL were improved. In the second one [23], Pt was supported on MoO_3_ and decorated by FeO_X_. The specific acidity of Pt-FeO_X_ interfaces was shown to be advantageous for the chemisorption and activation of C=O bond, promoting the selective hydrogenation of CAL to COL. In the present paper, we investigated the role of Mo-precursor on the catalytic behavior of Pt-Mo on carbon catalysts in cinnamaldehyde hydrogenation. We were able to demonstrate that the selectivity and activity of Pt-Mo catalysts are strictly connected with their physico-chemical properties, which in turn depended on both Mo precursor and heat treatment. A thermal pre-treatment of Mo/C has been shown to be beneficial in terms of activity, but the precursor of Mo appeared to be crucial for the selectivity of the reaction.

## 2. Materials and Methods

### 2.1. Catalyst Synthesis

Mo supported on carbon was synthesized by wet impregnation. The carbon support was Activated Charcoal Norit from Sigma-Aldrich. Two different catalysts were obtained by varying the Mo precursor, which was Na_2_MoO_4_ (Sigma Aldrich, St. Louis, MO, USA) or (NH_4_)_6_Mo_7_O_24_ (Sigma Aldrich), respectively. Mo loading was 10 wt % in both cases. The calculated amount of Mo salt precursor was dissolved in milli-Q water (100 mL/g of carbon). Then, carbon support was added, maintaining the solution under stirring. Impregnation lasted 4 h at room temperature (RT). After 4 h, temperature was increased at 80 °C, until the complete evaporation of the solvent. Half of the powder was used to support Pt nanoparticles (NPs) (labeled as fresh sample); the other half was submitted to thermal treatment at 400 °C in N_2_ flow (5 mL min^−1^) and then also used as support for Pt NPs (labeled as 400N sample). Pt NPs have been synthesized using sodium tetrachloroplatinate(II) hydrate (Na_2_PtCl_4_^●^3 H_2_O) as precursor. A 10 mg ml^−1^ of Na_2_PtCl_4_ solution was prepared first. One mL of the Na_2_PtCl_4_ solution was diluted in 50 mL of H_2_O (milli-Q), and then, 1 g of the Mo/C powder was added in the solution under continuous stirring. The amount of Mo/C was calculated to have a final 1 wt % Pt loading. The solution was kept under continuous stirring at RT for 2 h to impregnate the Pt^II^ salt on the Mo/C support. The powder was filtered and washed with distilled water; then, it was suspended again in 50 mL of H_2_O for the reduction of Pt. The reducing agent was NaBH_4_ (powder ≥ 98%, Sigma Aldrich). For such a step, NaBH_4_ was added directly under stirring, calculating the amount to have a NaBH_4_:metal molar ratio of 8:1. After 1 h, catalysts was filtered, washed, and dried at 80 °C for 2 h. Following the above procedure, four samples were prepared. The description and the corresponding labels are summarized in Table 1.

### 2.2. Characterization Methods

Transmission electron microscopy (TEM) characterization of the catalysts was performed by using a side entry JEOL 3010-UHR HRTEM microscope operating at 300 kV, equipped with a LaB_6_ filament, with a (2k × 2k)-pixel Gatan US1000 CCD camera and with an OXFORD INCA EDS instrument for atomic recognition via energy-dispersive spectroscopy (EDS). The powdered samples were deposited on copper grids, coated with a porous carbon film. A statistically representative number of particles (200–300 nanoparticles) was counted in order to obtain the Pt particle size distributions. The mean particle diameter (d_m_) was calculated using the following equation:

d_m_ = Σd_i_n_i_/Σn_i_

where n_i_ is the number of particles of diameter d_i_.

It is worth noting that as-prepared and treated samples were stable to prolonged exposition under the electron beam of the instrument (no metal coalescence nor modification of the Mo-activated carbon support).

X-ray photoelectron spectra (XPS) were taken in an M-probe apparatus (Surface Science Instruments) for the determination of surface composition and oxidation state of the metals. The source was monochromatic Al K radiation (1486.6 eV). Data reprocessing was performed by Esca Hawk software (Service physics Inc., Bend, OR, USA). The XPS lines of C 1s, O 1s, Mo 4f, and Mo 3d regions were recorded.

Ammonia Temperature Programmed Desorption (NH_3_-TPD) analyses were performed using a home-made gas-feeding apparatus described elsewhere [24] and connected downstream to a mass spectrometer (Hiden Analytical, HPR20, Warrington, UK). The samples were treated 130 °C under He flow for 1 h. Next, the temperature was lowered to 70 °C and ammonia was adsorbed by pulses of 100 μL until saturation. Then, the samples were purged at the same temperature with a 20 mL/min He flow. The desorption was performed by ramping up the temperature (8 °C/min) until about 550 °C.

### 2.3. Catalytic Reaction

Cinnamaldehyde hydrogenation has been carried out in a batch autoclave equipped with a glass inlet, at 80 °C and 5 bar of H_2_. The amount of the catalyst was calculated in order to have a metal:substrate molar ratio equal to 1:1000. The starting concentration of CAL was 0.15 M in 2-propanol. GC analyses performed by a Thermo Scientific TRACE 1300 Instrument (Waltham, MA, USA) equipped with an Agilent HP-5 column gave CAL conversion and products formation over time, using undecane as an external standard. Sampling occurred at time 0 and every 30 min, until the end of the reaction. The quantification of products was performed by calibrating the response with authentic samples.

## 3. Results and Discussion

Four samples (Table 1) consisting of 1 wt % Pt supported on 10 wt % Mo/C synthesized from (NH_4_)_6_Mo_7_O_24_ or Na_2_MoO_4_ have been prepared and tested in cinnamaldehyde hydrogenation (Table 2).

The carbon was first impregnated with the chosen precursor and then used as support for Pt NPs as it or treated at 400 °C in nitrogen. In the catalytic test, the cinnamaldehyde starting concentration was set to 0.15 M in 2-propanol, and the reaction occurred at 80 °C and 5 bar of H_2_ with a (Pt) metal/substrate molar ratio of 1000. The catalytic results are shown in Table 2. When Pt has been deposited on the fresh Mo/C, the catalysts did not show any activity (Table 2, entries 1 and 2), whereas when it has been deposited on the heat-treated Mo/C, the catalysts are active (Table 2, entries 3 and 4). Pt-Mo(Na)_400N achieved 66.4% conversion after 6 h of reaction, whereas Pt-Mo(NH_4_)_400N reached 50.8% (Table 2, entries 3 and 4). However, the initial activity of the Pt-Mo(Na)_400N was lower (330.9 h^−1^) than that of Pt-Mo(NH_4_)_400N (526.4 h^−1^) (Table 2).

**Figure 2:**
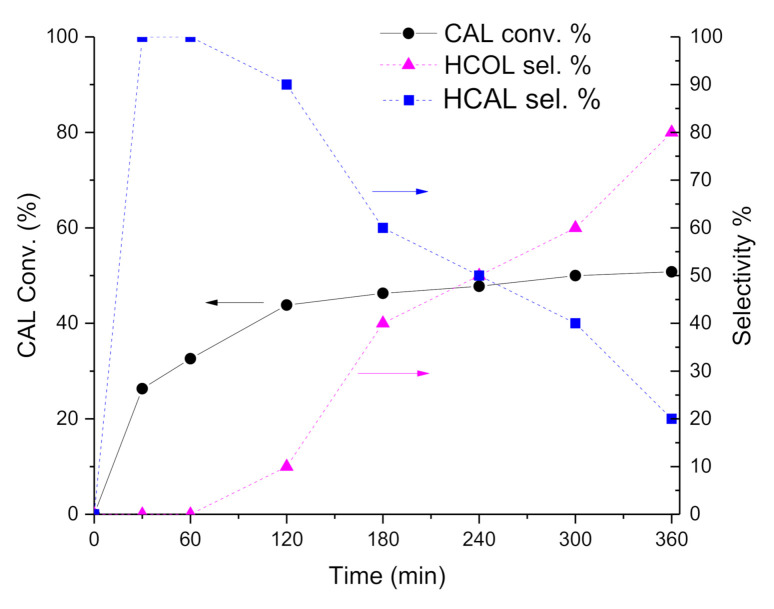
Cinnamaldehyde (CAL) hydrogenation reaction profile vs. time. CAL conversion % (solid line, left Y axis) along with hydrocinnamaldehyde (HCAL) and hydrocinnamyl alcohol (HCOL) selectivity % (dashed lines, right Y axis) over Pt-Mo(NH_4_)_400N.

**Figure 3:**
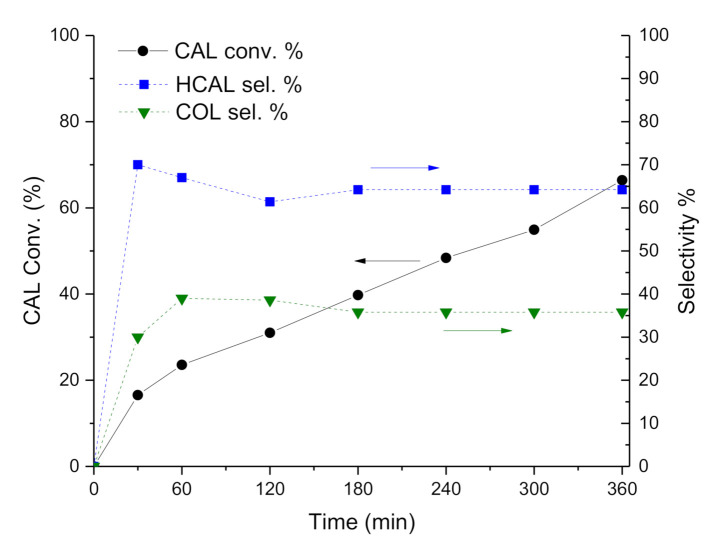
CAL hydrogenation reaction profile vs. time. CAL conversion % (solid line, left Y axis) along with HCAL and COL selectivity % (dashed lines, right Y axis) over Pt-Mo(Na)_400N.

In fact, reaction profiles and selectivity over time (Figure 2 and Figure 3) showed higher initial activity for Pt-Mo(NH_4_)_400N (Table 2 and Figure 2), but the conversion reaches a plateau after 3 h of reaction, which could point out a deactivation of the active sites. On the contrary, Pt-Mo(Na)_400N showed a constant increasing CAL conversion over time (Figure 3), despite the lower initial activity.

Concerning with selectivity, the Pt-Mo(NH_4_)_400N catalyst (Figure 2) initially converted CAL into HCAL quantitatively, but after 1 h of reaction (at about 30% of CAL conversion), HCAL started to be converted to HCOL, which reached a selectivity of 76.4% at 6 h of reaction (at 50.8% of CAL conversion). Differently, in the presence of Pt-Mo(Na)_400N (Figure 3), COL and HCAL seems to be formed contemporary, reaching a steady state, with a selectivity to COL and HCAL around 35% and 65%, respectively (see Table 2), regardless of the conversion.

Subsequently, we observed that depending on the Mo precursors, the catalysts showed very different catalytic performance: Pt-Mo(NH_4_)_400N presented a very fast hydrogenation of the double bond, which in turn did not allow obtaining COL. However, the catalyst was also able to hydrogenate the carbonyl group, thus producing HCOL from HCAL. On the contrary, Pt-Mo(Na)_400N was able to hydrogenate both groups with a more similar rate, thus producing COL + HCAL. However, HCAL is not converted to HCOL, meaning that the catalyst was not able to hydrogenate the CHO group if it is not conjugated with the double bond.

**Figure 4:**
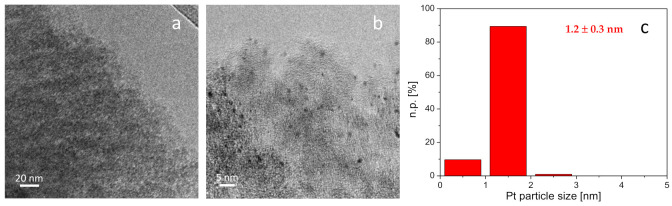
Representative TEM images of Pt-Mo(Na)_fresh (**a**) and Pt-Mo(Na)_400N (**b**). Pt particle size distribution of the sample treated at 400 °C in N_2_(**c**). Instrumental magnification: 100,000× and 250,000×, respectively.

Therefore, a detailed characterization has been carried out to explain the huge different catalytic behavior displayed by the Pt-Mo(Na)_fresh and Pt-Mo(NH4)_fresh catalysts (not active) and the corresponding Pt-Mo(Na)_400N and Pt-Mo(NH4)_400N catalysts (active). Moreover, these studies would be useful to establish structure–activity relationships by comparing Pt-Mo(Na)_400N and Pt-Mo(NH_4_)_400N catalysts. The TEM images of the Pt-Mo(Na)_fresh and the corresponding Pt-Mo(Na)_400N are reported in Figure 4a,b.

Only few very rare Pt nanoparticles have been observed in some regions of the sample when Pt has been deposited before the heat treatment (not shown), being both Mo and Pt highly dispersed on the support. EDS analysis revealed very weak peaks related to both Pt and Mo (Figure S1a). However, ICP analysis confirmed the actual loading of 1 wt % of Pt. A very low signal related to Na was also detected (0.11 wt %). On the contrary, when Pt has been deposited after the heat treatment of Mo/C, highly dispersed Pt nanoparticles with homogeneous shape and average size equal to 1.2 ± 0.3 nm were observed (Figure 3b,c). EDS analyses showed homogeneous distribution of both Pt (1.42 wt %) and Mo (0.30 wt %) (Figure S1b). In this case, a very low amount of Na (0.06 wt %) was found.

**Figure 5:**
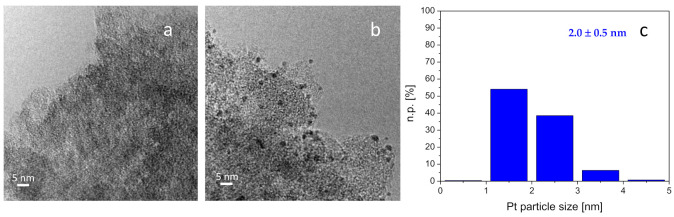
Representative TEM images collected on Pt-Mo(NH_4_)_fresh (**a**) and Pt-Mo(NH_4_)_400N (**b**). Particle size distribution of the sample treated at 400 °C in N_2_ (**c**). Instrumental magnification: 100,000× and 250,000×, respectively.

TEM-EDS analyses of the Pt-Mo(NH_4_)_fresh and Pt-Mo(NH_4_)_400N catalysts are reported in Figure 5. In addition, in this case, the Pt nanoparticles are not visible in the Pt-Mo(NH_4_)_fresh catalyst (Figure 5a), but both Mo (0.41 wt %) and Pt (0.14 wt %) were detected by the EDS probe. Similarly to what was observed for Pt-Mo(Na)_400N, Pt nanoparticles with average size of 2.0 ± 0.5 nm were observed in Pt-Mo(NH_4_)_400N (Figure 5b,c). EDS mapping showed that both Mo (1.61 wt %) and Pt (1.59 wt %) were homogeneously distributed on the support. In this case, also, some big Pt nanoparticle agglomerates were detected (Figure S2).

Then, it can be concluded that a role of the Mo precursor on the distribution of the Pt sites can be seen for Pt-Mo(Na)_400N and Pt-Mo(NH_4_)_400N. Indeed, it was observed that the size of the Pt particles is larger, and the particle size distribution is less homogeneous on the catalyst synthesized from (NH_4_)_6_Mo_7_O_24_ compared to that observed in the case of the one prepared using Na_2_MoO_4_ as the precursor. These differences in terms of size and distribution of Pt NPs could be connected to the differences created by the thermal treatment on (NH_4_)_6_Mo_7_O_24_/C and the Na_2_MoO_4_/C, as different Mo species or Mo–C interaction. This could also lead to a different Pt-Mo/C interaction.

Therefore, the surface of each catalyst was investigated by the XPS technique looking in particular at the Mo, Pt, and C species. The XPS survey analyses showing binding energies (B.E.) (eV) and the atomic % of Pt 4f and Mo 3d are reported in Table 3.

The Pt-Mo(Na)_fresh (Table 3, entry 1) does not show any signal of Mo, which is in agreement with EDS results. In the other cases, the B.E. showed similar results regardless of the heating treatment and the Mo precursor. The Pt-Mo(Na)_400 showed a Mo atomic percentage of 0.07 (Table 3, entry 2). Otherwise, the Pt-Mo(NH_4_)_fresh showed a Mo atomic percentage of 0.127% (Table 3, entry 3), which increased up to 0.48% in the Pt-Mo(NH_4_)_400 (Table 3, entry 4). Considering the Pt 4f, we found that there is a shift in the B.E. between the fresh and the heat-treated catalysts. Indeed, the B.E. shifted from 73.7 eV in the fresh (Table 3, entries 1 and 3) to 71.9 eV in the heat-treated (Table 3, entries 2 and 4). We found that Pt NPs deposited on different Mo/C samples showed different surface exposure, which is always higher in the heat-treated ones (see entries 2 and 4, Table 3). Moreover, looking at the XPS high-resolution spectra (Table 4, Table 5 and Table 6), some other interesting considerations can be done (images of the spectra are reported in the SI Figure S3). Also an XRD spectrum is reported in SI (Figure S4).

In Pt-Mo(Na)_fresh, no signal of Mo was revealed, while in the case of Pt-Mo(NH_4_)_fresh, we found a distribution of Mo^4+^ (232 eV) and Mo^6+^ (235 eV) of 55% and 44%, respectively. Concerning the heat-treated samples, the peak related to Mo 3d appeared in Pt-Mo(Na)_400.

From the deconvolution of the high-resolution spectrum, we found 44.2% of Mo^4+^ (232 eV) and 55.8% of Mo^6+^ (see Table 4, line 2) [25], which was different for Pt-Mo(NH_4_)_400N, showing 59% of Mo^4+^ and 40% of Mo^6+^ (Table 4). Considering the deconvolution of the high-resolution spectra of Pt 4f (Table 5), the peaks related to metallic Pt and PtO_x_ can be identified. As reported in the literature, the standard reference B.E. value for Pt^0^ is 71.0 eV [26], while slightly higher values can be attributed to the presence of partially oxidized Pt, as indicated as Pt^δ+^. PtO and PtO_2_ species show very similar binding energy values of 74.2 eV and 74.5 eV, respectively [27]. For this reason, it was rather difficult to discern between such two different oxidation states.

The deconvolution of the Pt-Mo(Na)_fresh spectrum showed two contributions of Pt: the first at 71.8 eV (46%) and the second at 75.2 eV (53%) (Table 5). We attributed the first to Pt^0/δ+^ and attributed the second to Pt^II/IV^. Comparing these data with the corresponding heat-treated Pt-Mo(Na)_400N sample, it was found that the B.E. of Pt^0/δ+^ is not changing so much, even if the percentage is double (from 46% to 82.5 at %); differently, the B.E. of Pt^II/IV^ decreased, thus showing a higher contribution of Pt^II^ species than Pt^IV^. In addition, the atomic percentage of oxidized species drastically decreased (from 53% to 17%). In the case of Pt-Mo(NH_4_)_fresh, the signal of Pt is very low and difficult to study. However, considering the Pt-Mo(NH_4_)_400N, we found that the B.E. attributed to Pt^0/δ+^ was 71.3 eV lower than Pt-Mo(Na)_400N, thus showing a higher contribution of Pt^0^ in this sample. The B.E. of Pt^II/IV^ was found at 73.0 eV, which was slightly lower than Pt-Mo(Na)_400N, meaning that the Pt^II^ species slightly increases compared to Pt-Mo(Na)_400N.

A different support functionalization was found to depend on the Mo/C used. Investigating the high-resolution spectra of carbon (Table 6), we interestingly found that upon heating, the C–O functionalization (285.4 eV) of the support always increased accompanied by a decrease of the amount of carboxylic groups (O−C=O at 288.0 eV). On the contrary, the C=O groups showed a different behavior, increasing in Pt-Mo(Na)_400N compared to Pt-Mo(Na)_fresh, while decreasing in Pt-Mo(NH_4_)_400N compared to Pt-Mo(NH_4_)_fresh.

On the basis of TEM and XPS analyses, we formulate a first hypothesis on the reason why fresh catalysts presented a negligible activity whilst the heat-treated samples resulted active, and, most importantly, why the Pt-Mo(NH_4_)_400N and Pt-Mo(Na)_400N behaved differently in terms of both activity and selectivity. Pt^0^ has been reported as the active site for hydrogenation of cinnamaldehyde [28,29], and it is also reported that the reduction of CHO group is favored by increasing particle size [29]; even the activity decreased.

These data could explain the higher activity of the heat-treated catalysts with respect to the fresh ones (see XPS data, Table 5), but they also presented discrepancies. Indeed, Pt-Mo(Na)_400N and Pt-Mo(NH_4_)_400N showed a similar Pt^0/δ+^ content (82% and 78%) and slightly different particle size (1.2 and 2.0 nm), which could be a consequence of the higher content of O-containing functionalities in the case of Pt-Mo(Na)_400N than Pt-Mo(NH_4_)_400N (Table 6). However, Pt-Mo(NH_4_)_400N showed a higher initial activity (Table 2) compared to Pt-Mo(Na)_400N, but it underwent to deactivation phenomena and presented a selectivity toward a full hydrogenated product, i.e., HCOL.

Then, we considered the presence of different species of Mo, i.e., Mo^IV^ and Mo^VI^ as evidenced by XPS (Table 4). It was reported that the B.E. of Pt is reported to shift at lower value when Pt interacts with MoO_x_ [30]. Looking at the XPS data, we observed a lower B.E. for Pt in the case of Pt-Mo(NH_4_)_400N (Table 5) (and a higher presence of MoO_2_), thus confirming the higher reduction of Pt compared to Pt-Mo(Na)_400N. At the opposite, MoO_3_ is reported to stabilize a more positive state of Pt [31] that can explain the high B.E. of Pt (72.2 eV, Table 5) in Pt-Mo(Na)_400N.

Summarizing, we can conclude that Mo precursors direct the functionalization of C during the calcination step and form a different ratio of MoO_2_ and MoO_3_. These different species have a different impact in the dispersion of Pt and in its final oxidation state, which in turn modify the catalytic activity. However, even explaining the different activity, these findings cannot account for the different selectivity shown by the two catalysts. Thus, we search the explanation in a possible different acidity, as it is reported [32] that this parameter can have a decisive role in the hydrogenation rates of C=C/C=O.

**Figure 6:**
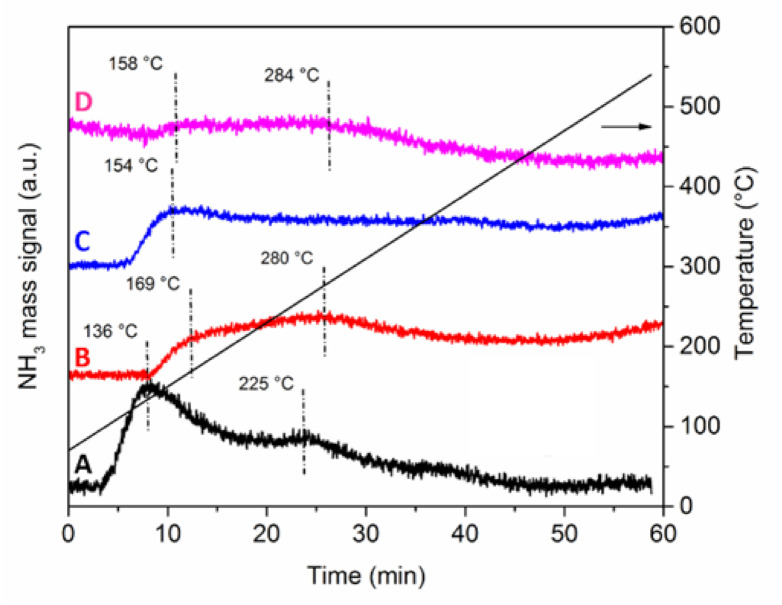
Ammonia Temperature Programmed Desorption (NH_3_-TPD) profiles of the Pt-Mo(NH_4_)_fresh (**A**) and Pt-Mo(NH_4_)_400N (**B**) samples, and of the Pt-Mo(Na)_fresh (**C**) and Pt-Mo(Na)_400N (**D**) samples.

Acid sites strength was investigated by NH_3_-TPD analyses (Figure 6). The quantification of the acid sites was very difficult, because not all the profiles returned to the baseline level at high temperature. However, some qualitative considerations can be drawn.

Both Pt-Mo(NH_4_)_fresh (sample A) and Pt-Mo(Na)_fresh (sample C) displayed desorption profiles with two main components, which is indicative of acid sites with different acid strength. The low-temperature component below 160 °C can be attributed to acid sites with low strength [33], while the broad feature at higher temperature can be due to highly dispersed Pt-Mo sites with medium acid strength [7].

The corresponding Pt-Mo(NH_4_)_400N (sample B) and Pt-Mo(Na)_400N (sample D) have similar profiles, which are qualitatively less intense than the previous ones, but with both components at slightly higher desorption temperatures. Thus, the treatment in N_2_ at 400 °C influenced the acidity of the samples. Moreover, there is a difference among the two thermally treated catalysts in the low-temperature region: the sample prepared from (NH_4_)_6_Mo_7_O_24_ shows a component at 169 °C, whereas the one synthesized from Na_2_MoO_4_ is at 158 °C. Such difference in desorption temperature suggests that the Pt-Mo(NH_4_)_400N catalyst has stronger acid sites compared to the Pt-Mo(Na)_400N sample.

It has been reported that a decrease in acidity results in greater electron density on the metal particles [34], which may cause a suppression in the C=C hydrogenation by enhancing the delocalization of electrons in the adsorbed conjugated substrate.

Even if Pt usually activates the C=O group [17,18], a more acidic Pt catalyst has poorer electron density, which reduces the electron repulsion related to the C=C adsorption, favoring the formation of hydrocinnamaldehyde [32]. Thus, Pt-Mo(NH_4_)_400N mostly produced HCAL due to the presence of more acidic sites, resulting in a catalyst selective toward C=C hydrogenation.

## 4. Conclusions

Two different Mo precursors have been used to modify the active carbon used as support for Pt NPs. The thermal treatment of Mo/C before the Pt deposition resulted in an enhanced exposure of Pt, high NP dispersion and in higher reduction, thus promoting the catalytic activity. We ascribed this behavior to the different functionalization of carbon support, which in turn affects the MoO_2_/MoO_3_ ratio (XPS). However, the Pt-Mo(Na)_400N and Pt-Mo(NH_4_)_400N samples behaved differently not only from an activity point of view, but also from that of selectivity. In particular, Pt-Mo(NH_4_)_400N produced selectively HCAL, which subsequently underwent hydrogenation to HCOL. On the contrary, Pt-Mo(Na)_400N is able to hydrogenate C=C and C=O with an almost comparable rate, which results in a 65:35 mol/mol ratio of HCAL:COL. Acidity studies revealed that another effect produced by the different Mo precursors is to vary the strength of acid sites on the surface, Pt-Mo(NH_4_)_400N presenting stronger acid sites compared to Pt-Mo(Na)_400N. On the basis of the literature, we then ascribed the selectivity of Pt-Mo(NH_4_)_400N toward C=C hydrogenation to the higher acidity of this catalyst with respect to Pt-Mo(Na)_400N.

## Figures and Tables

**Table 1:** Samples list and description.

n.	Sample	Description
1	Pt-Mo(NH_4_)_fresh	Pt NPs on active carbon impregnated with (NH_4_)_6_Mo_7_O_24_.
2	Pt-Mo(NH_4_)_400N	Pt NPs on thermally treated (400 °C in N_2_) active carbon impregnated with (NH_4_)6Mo_7_O_24_.
3	Pt-Mo(Na)_fresh	Pt NPs on active carbon impregnated with Na_2_MoO_4_.
4	Pt-Mo(Na)_400N	Pt NPs on thermally treated (400 °C in N_2_) active carbon impregnated with Na_2_MoO_4_.

**Table 2:** Catalytic results of cinnamaldehyde hydrogenation by Pt-Mo catalysts.

n.	Catalyst	In. Activity [a]	Conv. % (6 h) [b]	HCAL Sel.% [c]	HCOL Sel.%	COL Sel.%
1	Pt-Mo(Na)_fresh	0	0	-	-	-
2	Pt-Mo(NH_4_)_fresh	0	0	-	-	-
3	Pt-Mo(Na)_400N	330.9	66.4	64.3	-	35.7
4	Pt-Mo(NH_4_)_400N	526.4	50.8	23.6	76.4	-

Reaction conditions: CAL 0.15 M in 2-propanol; 80 °C; 5 bar of H2; metal:substrate molar ratio 1000. [a] Initial activity calculated at 30 min of reaction, as moles converted per moles of metal. [b] Conversion calculated after 6 h, as percentage of moles of converted substrate. [c] Selectivity calculated as percentage of by-products formed.

**Table 3:** XPS survey analyses.

			XPS Line			
			C 1s	O 1s	Pt 4f	Mo 3d
*n.*	*Catalyst*					
1	Pt-Mo(Na)_fresh	*B.E. (eV)*	*284.6*	*532.7*	*73.7*	*nd*
		**At %**	**88.9**	**9.7**	**0.01**	**0**
2	Pt-Mo(Na)_400N	*B.E. (eV)*	*284.6*	*532.7*	*71.9*	*231.9*
		**At %**	**92.7**	**7.1**	**0.11**	**0.1**
3	Pt-Mo(NH_4_)_fresh	*B.E. (eV)*	*284.6*	*532.6*	*73.7*	*232.9*
		**At %**	**90.4**	**9.0**	**n.d.**	**0.13**
4	Pt-Mo(NH_4_)_400N	*B.E. (eV)*	*284.6*	*532.5*	*71.9*	*232.5*
		**At %**	**90.5**	**8.3**	**0.23**	**0.48**

**Table 4:** High-resolution X-ray photoelectron spectroscopy (XPS) analyses of Mo 3d region.

		Mo 3d	
Sample		Mo^4+^	Mo^6+^
Pt-Mo(Na)_fresh	*B.E. (eV)*	*nd*	*nd*
	**At %**	-	-
Pt-Mo(Na)_400N	*B.E. (eV)*	*232.0*	*235.0*
	**At %**	**44.2**	**55.8**
Pt-Mo(NH_4_)_fresh	*B.E. (eV)*	*232.0*	*235.0*
	**At %**	**55**	**44**
Pt-Mo(NH_4_)_400N	*B.E. (eV)*	*232.7*	*235.8*
	**At %**	**59**	**40**

**Table 5:** High-resolution XPS analyses of the Pt 4f region.

		Pt 4f	
Sample		Pt^0/δ+^	Pt^II/IV^
Pt-Mo(Na)_fresh	*B.E. (eV)*	*71.8*	*75.2*
	**At %**	**46**	**53**
Pt-Mo(Na)_400N	*B.E. (eV)*	*72.2*	*73.4*
	**At %**	**82.5**	**17.5**
Pt-Mo(NH_4_)_fresh	*B.E. (eV)*	*nd*	*nd*
	**At %**	**-**	**-**
Pt-Mo(NH_4_)_400N	*B.E. (eV)*	*71.3*	*73.0*
	**At %**	**78**	**22**

**Table 6:** High-resolution XPS analyses of the C 1s region.

		C 1s			
sample		C−C/C=C	C−O	C=O	O−C=O
Pt-Mo(Na)_fresh	*B.E. (eV)*	*284.6*	*285.4*	*286.9*	*288.8*
	**At %**	51.8	30.9	6.2	11.0
Pt-Mo(Na)_400N	*B.E. (eV)*	*284.6*	*285.3*	*286.3*	*288.0*
	**At %**	32.1	43.8	13.8	6.8
Pt-Mo(NH_4_)_fresh	*B.E. (eV)*	*284.6*	*285.8*	*287.5*	*289.6*
	**At %**	65.6	23.2	5.3	5.9
Pt-Mo(NH_4_)_400N	*B.E. (eV)*	*284.6*	*285.5*	*286.9*	*288.1*
	**At %**	59.5	28.8	3.7	4.4

## Data Availability

Not applicable.

## Supplementary Materials

The following are available online at https://www.mdpi.com/2079-4991/11/2/362/s1, Figure S1: EDS spectra of 1 wt % Pt-10 wt % Mo/C from Na_2_MoO_4_ before (a) and after (b) thermal treatment at 400 °C in N_2_. Figure S2: TEM image collected on the Pt-Mo(NH_4_)_400N sample and corresponding EDS map of the same region showing the relative location of Pt, Mo, C and O. Instrumental magnification: 20,000×. Figure S3: XPS spectra. Figure S4: XRD spectrum.
