# Supplementary material for: Ruling Factors in Cinnamaldehyde Hydrogenation: Activity and Selectivity of Pt-Mo Catalysts
Source: Nanomaterials (Basel). 2021 Feb 1;11(2):362. doi: 10.3390/nano11020362 (PMC7912768; doi:10.3390/nano11020362)
Supplement: Supplementary file 1 [file nanomaterials-11-00362-s001.pdf]

Supporting information

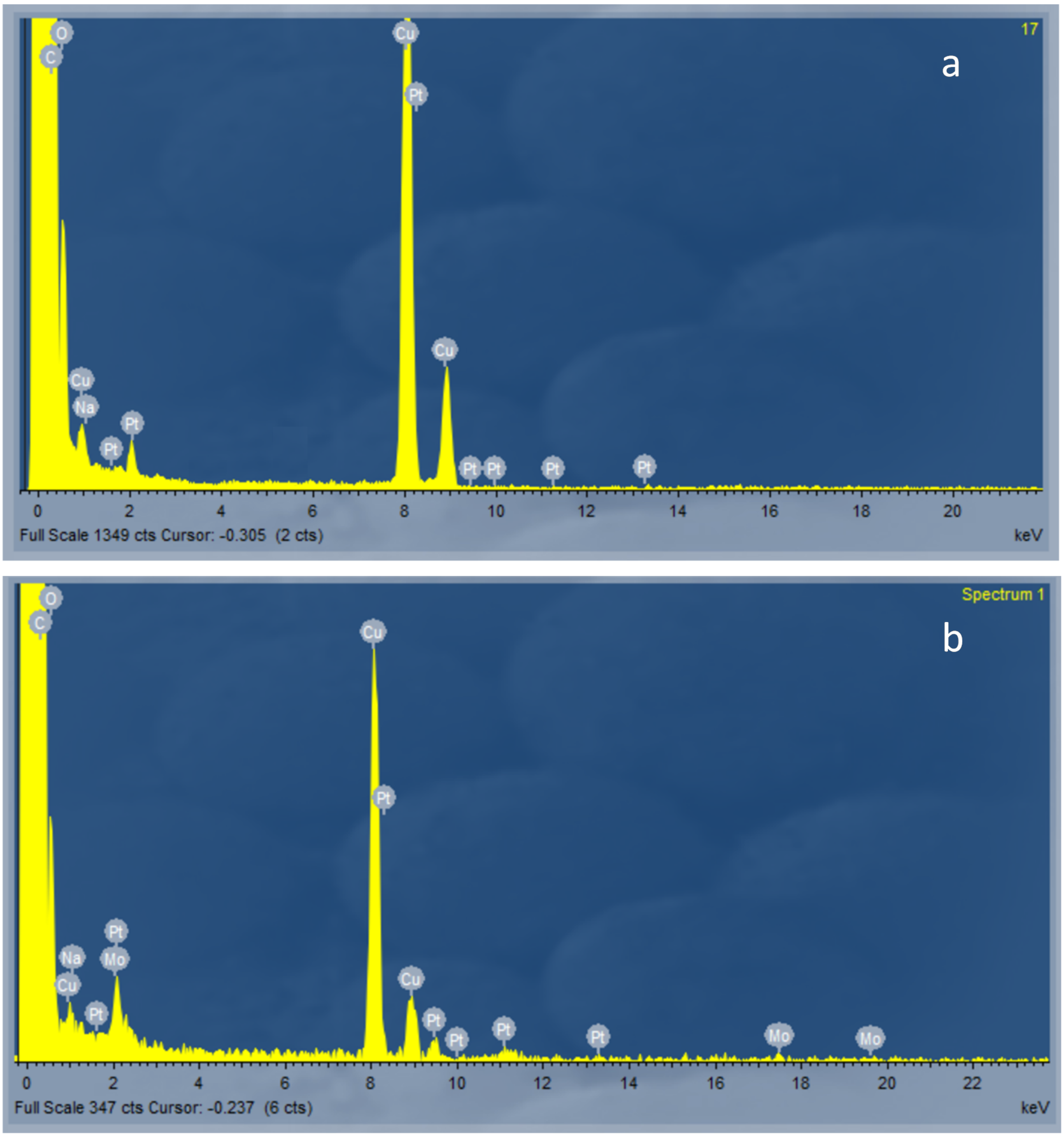

Figure S1. EDS spectra of 1 wt% Pt-10 wt% Mo/C from Na_2MoO_4 before (a) and after (b) thermal treatment at 400 °C in N_2.

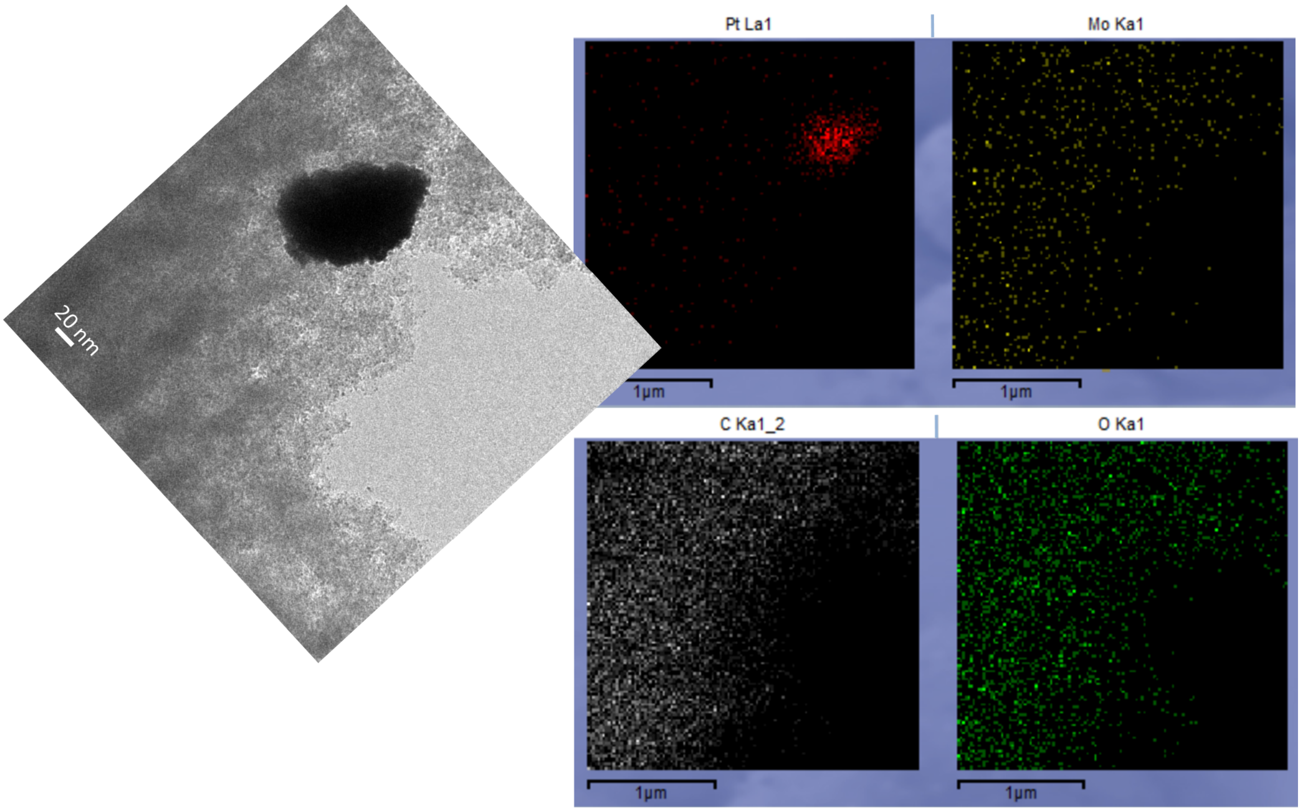

Figure S2. TEM image collected on the Pt-Mo(NH_4)_400N sample and corresponding EDS map of the same region showing the relative location of Pt, Mo, C and O. Instrumental magnification: 20000×.

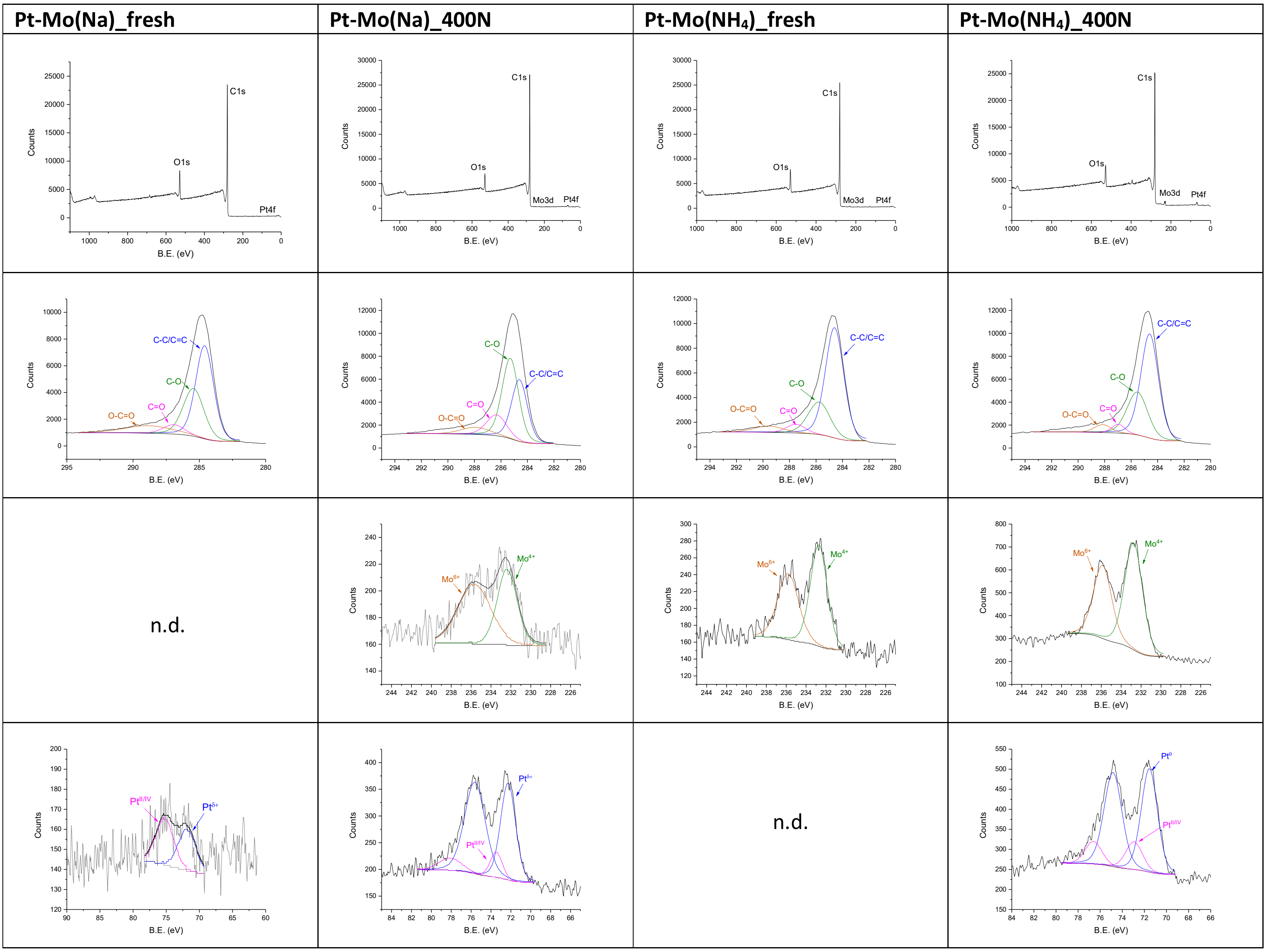

Figure S3. Complete XPS high-resolution spectra of the catalysts

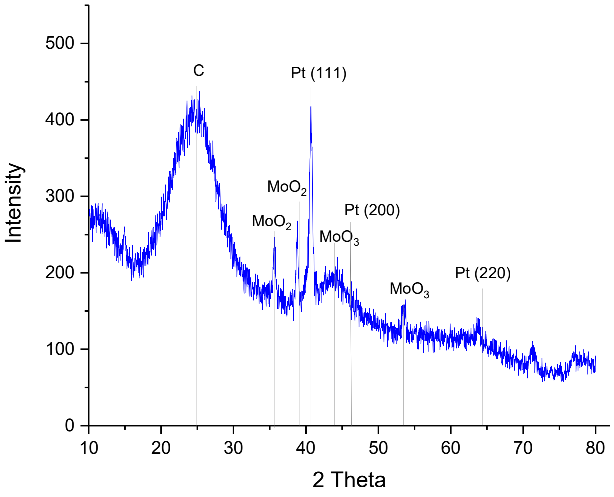

Figure S4. XRD of heat treated Pt-Mo supported on carbon catalyst.
